# Prevalence and incidence of cognitive impairment following acute respiratory distress syndrome of any cause: a systematic review and meta-analysis

**DOI:** 10.1186/s13054-025-05375-x

**Published:** 2025-04-23

**Authors:** Robert Pohl, Doreen Wolff, Ebru Özkan, Antonia A. Sprenger, Claudia Hasenpusch, Judith Wesenberg, Emrah Düzel, Christian Apfelbacher

**Affiliations:** 1 Institute of Social Medicine and Health Systems Research, Otto-von-Guericke University Magdeburg, Magdeburg, Germany; 2 Institute of Cognitive Neurology and Dementia Research, Otto-von-Guericke University Magdeburg, Magdeburg, Germany

**Keywords:** Cognitive impairment, Acute respiratory distress syndrome (ARDS), Prevalence, Meta-analysis

## Abstract

**Objectives:**

The aim of this systematic review and meta-analysis is to synthesize and appraise the evidence on prevalence of cognitive impairment following acute respiratory distress syndrome (ARDS) of any cause.

**Methods:**

We systematically searched PubMed, Scopus, and Web of Science for observational studies focused on cognitive impairment in adult survivors of ARDS. Risk of bias and certainty of evidence (GRADE) were assessed. A meta-analysis using a random effects model was performed to estimate the overall prevalence of cognitive impairment after ARDS, with subgroup analyses for COVID-19-related ARDS (C-ARDS). Additionally, a meta-regression was conducted to assess the influence of demographic and clinical predictors on cognitive outcomes. Heterogeneity was assessed using τ^2^ and the I^2^ statistic.

**Results:**

We identified 14 studies with 1451 participants, with 650 participants (range: 13–98) included in the analyses. In the subgroup of C-ARDS, 12 studies with 563 participants (range: 13–98) were considered. The pooled prevalence of cognitive impairment following ARDS was 36% (95% CI 26–46%), with high heterogeneity between studies (I^2^ = 92%, τ^2^ = 0.03). In C-ARDS cohorts, the prevalence was 34% (95% CI 22–45%), with similar levels of heterogeneity (I^2^ = 92.7%, τ^2^ = 0.03). Meta-regression analysis showed  that older age predicted a higher prevalence of cognitive impairment following ARDS (b = 0.02, p = 0.033), reducing between-study heterogeneity (I² = 60.04%, τ² = 0.01). ICU stay, sex, and time from ICU discharge to cognitive assessment showed no significant associations (p > 0.05).

**Conclusions:**

This meta-analysis corroborates previous findings that cognitive impairment remains a persistent issue for ARDS survivors. The prevalence of cognitive impairments following ARDS highlights the importance of future research to unravel the complex underlying mechanisms contributing to these deficits and to develop targeted strategies for prevention and rehabilitation in survivors.

**Supplementary Information:**

The online version contains supplementary material available at 10.1186/s13054-025-05375-x.

## Introduction

Acute respiratory distress syndrome (ARDS) is characterized by diffuse lung damage, which can result from a wide range of pulmonary and extrapulmonary conditions. Individuals with ARDS experience sudden severe respiratory distress and hypoxemia, making ARDS one of the key criteria for intensive care unit (ICU) admission [1, 2]. As a result, ICU cohorts are frequently studied in observational research focussed on long-term morbidity following ARDS. ARDS represents 0.42 cases per ICU bed over 4 weeks, accounting for 10.4% of ICU admissions and 23.4% of patients requiring mechanical ventilation [3]. According to the LUNG SAFE study (2016), 10.4% of ICU admissions result in ARDS, representing 23.4% of all invasively ventilated patients. Pneumonia (59.4%), extrapulmonary sepsis (16%), and aspiration (14%) are the most common causes of ARDS [3].

Survivors of ARDS face a heightened risk of long-term comorbidities [4], reduced quality of life [5], and physical and mental health impairments [6]. Furthermore, they contribute to increased healthcare costs [7]. One of the most frequent and debilitating consequences of ARDS is cognitive impairment, which can be severe and persistent [8, 9]. Several systematic reviews have investigated health-related quality of life, as well as physical, and psychological impairments in ARDS survivors [6], explored the pathophysiological mechanisms behind cognitive impairment after ARDS [10], and, in part, assessed the prevalence of cognitive impairment after ICU [11]. However, a comprehensive review of the prevalence and incidence of cognitive impairment post-ARDS is still lacking, leaving an incomplete understanding of the long-term cognitive impact on ARDS survivors.

Moreover, existing systematic reviews [10–12] largely rely on data from pre-COVID-19 studies, yet the incidence of ARDS has significantly increased since the onset of the SARS-CoV-2 pandemic. A narrative review by Riordan et al. (2020) was one of the few early studies to address neuropsychological outcomes in acute respiratory diseases, highlighting the potential for cognitive impairment in COVID-19 recovery [13]. The underlying mechanisms linking ARDS to cognitive impairment are complex and may involve inflammation, hypoxia, and neurological damage. The identification and consideration of pre-existing cognitive deficits pose a significant challenge for clinical management and recruitment into longitudinal studies, as described by Sasannejad et al. (2019) [10].

This emphasizes the complex and multifactorial relationship between ARDS and cognitive impairments, including mild cognitive impairment (MCI). These findings underscore the pressing need for further research to establish definitive conclusions regarding the prevalence and incidence of cognitive impairment following ARDS. Thus, the aim of this systematic review and meta-analysis is to systematically summarize and appraise the evidence on prevalence and incidence rates of cognitive impairment following ARDS, irrespective of the underlying cause. Based on the existing literature, we hypothesize that cognitive impairment is a frequent and significant long-term consequence of ARDS, with a considerable prevalence and incidence among survivors. Given the multifactorial nature of ARDS-related cognitive decline, we expect to observe variations in prevalence depending on demographic factors, clinical factors, and assessment methods.

## Methods

The guidelines outlined in the Preferred Reporting Items for Systematic Reviews and Meta-Analyses (PRISMA) and the Meta-analysis of Observational Studies in Epidemiology (MOOSE) checklist guided the reporting of this systematic review and meta-analysis. The protocol for this review was registered with the International Prospective Register of Systematic Reviews and Meta-analysis (PROSPERO): CRD42024524476.

### Search strategy and study selection

We systematically searched PubMed, Scopus, and Web of Science for observational studies published in German or English between 2013 and March 12, 2024.The primary outcome of this study was cognitive impairment, assessed based on prevalence estimates reported in the included studies. We extracted information on the measurement instruments, cognitive scores, and timing of assessment where available. However, the specific tests or assessment time points were not used as inclusion or exclusion criteria, as our objective was to provide a comprehensive synthesis of prevalence data across different studies, regardless of variations in assessment methods. A manual search was conducted using the reference lists of full-text articles using Spidercite from the Systematic Review Accelerator Guidelines. The search strategy was updated on August 30, 2024, immediately prior to the final analysis to include the most recent and relevant studies. The full search strategy is available in Supplemental Content (SC) 1.

The inclusion criteria followed the PECO-S (population, exposure, comparator, outcome, study design) framework and encompassed observational studies—including cross-sectional, cohort (prospective and retrospective), and case control designs that reported cognitive impairment in adult (≥ 18 years) survivors of ARDS. Both subjective cognitive impairment and MCI were considered. Studies with paediatric or adolescent patients, interventional studies (e.g., randomized controlled trials), quasi-experimental designs, qualitative research, case series, and studies focused on patients with pre-existing neurological conditions (e.g., dementia, Alzheimer’s disease, traumatic brain injury, or stroke) were excluded. Additional exclusion criteria comprised studies on patients undergoing chemotherapy, post-intensive care syndrome without ARDS, and those investigating delirium or postoperative cognitive dysfunction (POCD) exclusively.

Titles, abstracts, and full texts of each record were screened by three independent reviewers (RP, DW, EÖ) following the inclusion criteria. Discrepancies were resolved through discussion or consultation with a fourth reviewer if necessary. In full-text screening, a key inclusion criterion was the precise description of ARDS and its association with the outcome of cognitive impairment. The details of the excluded studies are in the SC 6.

Results of each search were merged into one single file in CITAVI 27 (Swiss Academic Software), which was uploaded to Rayyan for duplicate removal as well as title, abstract, and full-text screening.

Detailed inclusion and exclusion criteria for title and abstract screening, including guidelines for conducting the full-text review, are available in the supplemental content (SC2, SC3).

### Data extraction

Extracted data included information on the primary outcome (prevalence and incidence of cognitive impairment among ARDS survivors), study characteristics (study design, data collection period, aim, inclusion and exclusion criteria, location, population, disease-related, demographic details), and details about cognitive assessment (e.g., instruments used and timing of assessments in months post-ICU admission). Mechanical ventilation, ICU stay, and COVID-associated ARDS as well as information required for risk of bias and certainty of evidence assessments were additionally extracted.

### Risk of bias and certainty of evidence assessment

Given the observational nature of most eligible studies, the risk of bias was assessed independently by two reviewers (RP, DW) using the Newcastle–Ottawa Scale (NOS) [14]. The NOS assesses the quality of design of nonrandomized studies and consists of eight items, divided into three broad criteria: selection, comparability, and either outcome (in cohort studies) or exposure (in case–control studies) [14]. The NOS helps evaluate key aspects of the study design, such as participant selection, comparability of groups, and outcome assessment, which are critical for understanding the reliability of the results. However, the NOS has limitations, as it may not fully capture all potential sources of bias, such as confounding variables or incomplete reporting. Additionally, its reliance on subjective judgment in certain areas can introduce variability in the quality assessment, which may affect the interpretation of the findings.

The certainty of evidence for each outcome was evaluated by two independent reviewers using the Grading of Recommendations, Assessment, Development and Evaluations (GRADE) approach. We used GRADEpro to support us in the GRADE assessment [15].

### Statistical analysis

The characteristics and results of all included studies were summarized descriptively and all studies were considered for quantitative synthesis.

A meta-analysis of the overall prevalence rates of cognitive impairment after ARDS was conducted using a random-effects model with τ^2^ estimated by the restricted maximum likelihood (REML). Results were presented in forest plots, annotated with heterogeneity statistics and p-values. As measures of heterogeneity, τ^2^ and the *I*^2^ statistic were reported along with their respective confidence intervals, using a rough interpretation of the *I*^2^ statistics (low: 0–25%, moderate: 25–75%, high: 75–100%) [16]. Egger’s regression test and funnel plots were used to detect potential publication bias [17]. Subgroup analysis was conducted in studies reporting exclusively on cognitive impairment after C-ARDS. To examine the robustness of the pooled estimates, a further subgroup analysis was performed in studies that used the Montreal Cognitive Assessment (MoCA) tool.

To assess potential multicollinearity, correlations between all predictors of the meta-regression were examined, including demographic (average patient age and proportion of male patients) and clinical predictors (time between ICU discharge and cognitive assessment, ICU length of stay, and duration of mechanical ventilation). ICU length of stay and the duration of mechanical ventilation were highly correlated (r = 0.89). To avoid potential multicollinearity, mechanical ventilation duration was not included in the meta-regression. The remaining predictors showed weaker associations, with all correlation coefficients being less than or equal to 0.65. Meta-regressions were thus conducted to assess the association of time between of ICU discharge and cognitive assessment, ICU length of stay, age, and sex with cognitive impairment. We first performed simple meta-regressions for each predictor and subsequently entered all predictors into the meta-regression model to account for potential confounding. Additionally, bubble plots were used to illustrate associations observed across studies. Statistical analyses were performed using R free software for statistical computing [18] version 4.4.2 and the R package ‘metafor’ [19].

## Results

### Characteristics of the included studies

**Fig. 1:**
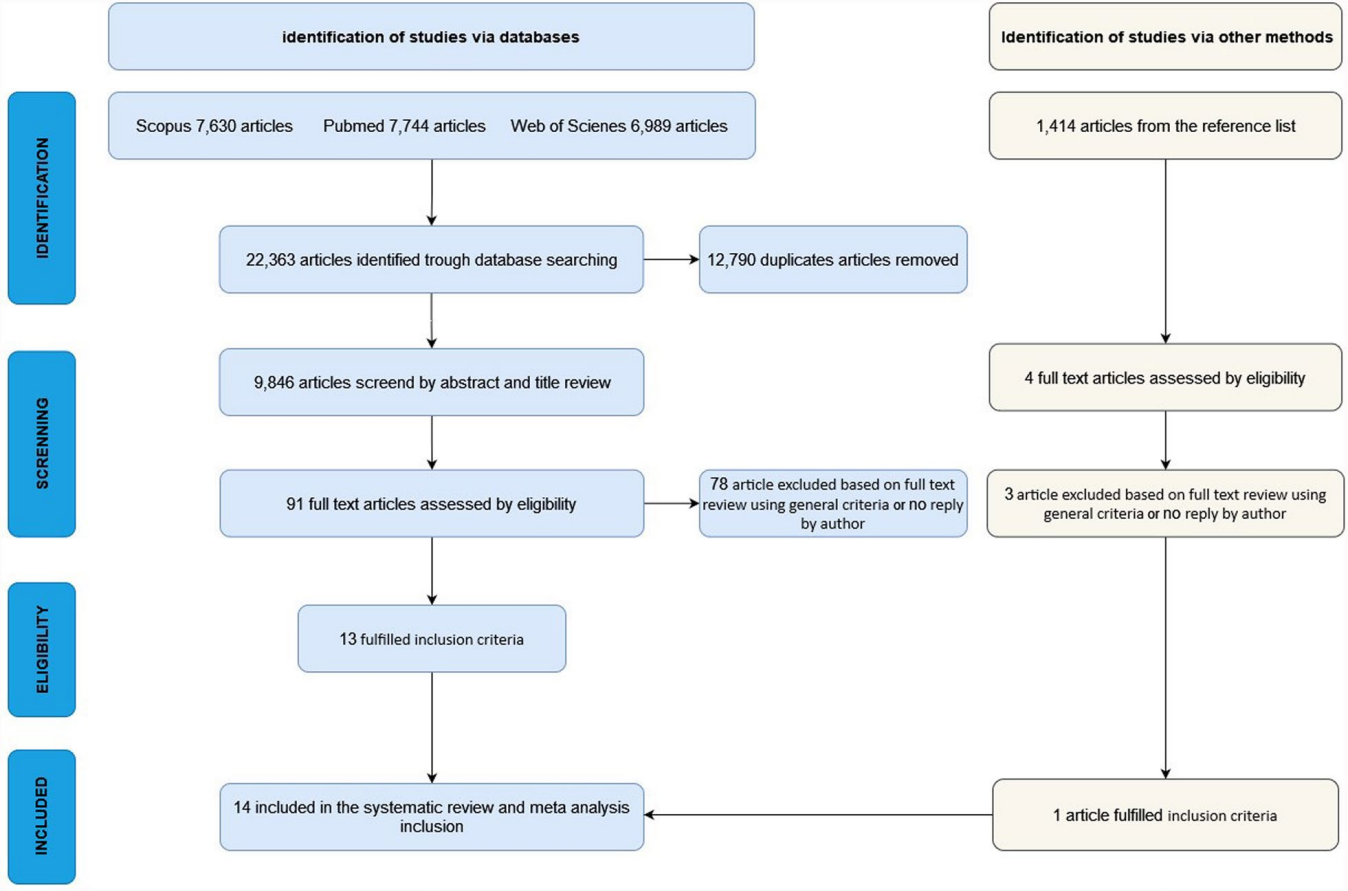
Search methodology and selection process

The search strategy identified a total of 22,363 publications across three different databases. After removing duplicates, 9846 titles and abstracts were screened for eligibility. Of these, 91 studies were selected for full-text evaluation, with 74 subsequently excluded after detailed assessment. The most common reasons for exclusion were data collection predating 2013 (often related to the ARDSNet Long-Term Outcomes Study) or insufficient description of ARDS. A detailed list of exclusion criteria is provided in SC 4. Additionally, 1414 studies were screened based on the reference lists of the 91 full-text studies, from which one additional study was included in the analysis. In total, 14 studies were included in the systematic review and meta-analysis. The flowchart of this process is presented in Fig. 1.

The study characteristics are summarized in Table 1. The included studies encompass a total of 1451 adult ARDS survivors treated in intensive care units. Among the 14 studies, all are single-centre; two are longitudinal, 11 are prospective observational studies, and one employs a cross-sectional design. The studies were conducted in various countries across four continents, including Japan, the United States, Italy, Belgium, France, and others. Eleven studies report on C-ARDS patients, with one additional study comparing C-ARDS to non-C-ARDS cohorts [20]. Table 2 summarises the characteristics and results of the study samples with cognitive impairment.

**Table 1:** Characteristics of all studies included in the systematic review

Study	Country	Design	Sample size	Population
Beaud et al. [21]	Switzerland	Cross sectional study	13	C-ARDS cohort
Okazaki et al. [22]	Japan	Prospective observational study	192	PICS assessment cohort (sepsis and non-sepsis group; ARDS and non-ARDS subgroups)
Pozzi et al. [23]	Italy	Prospective observational study	243	C-ARDS cohort with ECMO (patients were stratified by follow-up vs. lost at follow-up)
Maley et al. [24]	USA	Prospective observational study	63	C-ARDS who require mechanical ventilation
Martinez et al. [25]	Argentina	Prospective Observational Study	52	C-ARDS survivors who required mechanical ventilation
Monti et al. [26]	Italy	Prospective observational study	65	Invasively ventilated C-ARDS survivors
Weidman et al. [27]	USA	Retrospective observational study	124 (main cohort), 1383 (comparison cohort)	Main cohort: C-ARDS ICU survivors with appointment at Weill Cornell Medicine’s (WCM) post-ICU recovery clinic; comparison cohort: same characteristics but without attendance of post-ICU clinic
Sylvestre et al. [28]	France	Prospective observational study	40	ECMO and non-ECMO patients with ARDS
Rubega et al. [29]	Italy	Prospective observational study	91	C-ARDS survivors
Sturgill et al. [20]	USA	Retrospective observational study	94	C-ARDS and non- C-ARDS survivors
Vanginderhuysen et al. [30]	Belgium	Prospective observational study	85	C-ARDS survivors
Duindam et al. [31]	The Netherlands	Prospective longitudinal study	96	C-ARDS survivors
Jaquet et al. [32]	France	Prospective observational Study	280	C-ARDS survivors
Latronico et al. [33]	Italy	Prospective longitudinal study	137	C-ARDS survivors

**Table 2:** Characteristics of included studies

Study	Participants	Mechanical ventilation (days)	ICU stay (days)	Cognition test used	ICU discharge to cognitive assessment	Extracorporeal membrane oxygen, n (%)	pO_2_/FiO_2_ ratio, mmHg	ARDS severity	Prevalence of cognitive impairment in percent
Beaud et al. [21]	n = 13, mean age: 64.8 (± 7.6), 76.9% male	M (SD) = 21.5 (11.2)	M (SD): 28.10 (15.2)	MoCa	M (SD): 5.5 (2.4) days	Not described	Not described	Severe n = 13	69.2
Okazaki et al. [22]	n = 17, median age: 70 (64–77), 58.8% male	Median (IQR) = 6 (4–8)	Median (IQR): 8 (6–12)	Short Memory Questionnaire (SMQ)	6 months	n = 3	Not described	Not described	41.2 (Incidence)
Pozzi et al. [23]	n = 18, median age: 51 (38–55), 64% male	Median (IQR): 25 (19–50)	Median (IQR): 28 (21–49)	MoCa	Median (IQR): 96 (78–128) days	n = 18	69 (52–87)	Not described	29
Maley et al. [24]	n = 60, mean age: 59 (± 13), 48.3% male	M (SD): 15.6 (± 5.1)	Not described	MoCA-Blind	M (SD): 182 (15) days	n = 2	164 (± 42), mean at intubation	Not described	42
Martinez et al. [25]	n = 22, median age: 68 (58–71), 68% male	Median (IQR): 37 (12–46)	Median (IQR): 43 (21–56)	MoCa	Median (IQR): 59 (50–60) days	Not described	Not described	Severe n = 6	41
Monti et al. [26]	n = 39, mean age: 56 (± 10.5), 90% male	Median (IQR): 9 (6–14)	Median (IQR): 10 (7–16)	Italian telephonic version of the Mini-Mental State Examination (Itel-MMSE)	Median (IQR): 61 (51–71) days	n = 2	125 (± 61.8)	Mild n = 4; Moderate n = 18; Severe n = 15; 2 missings	2.6
Weidman et al. [27]	n = 59, mean age: not described in MoCa group, no % sex in MoCa group described	Not described in MoCa group	Not described in MoCa group	MoCA & MoCA-Blind	20 days	Not described	Not described	Not described	25
Sylvestre et al. [28]	Non-ECMO-group (n = 22, median age: 51 (43–63), 61% male); ECMO-group (n = 18, median age: 41 (32–56, 55% male)	Non-ECMO-group median (IQR): 29 (21–46); ECMO-group median (IQR): 36 (28–64)	Non-ECMO-group median (IQR): 35 (24–47); ECMO-group median (IQR): 46 (34–71)	Wechsler Adult Intelligence Scale, 4th edition (WAIS-IV)	Median (IQR): 20 (17–22) and 22 (18–23) months	n = 22	Lowest PaO₂ at Day 1, mmHg: 71.2 mmHg; Duration of Hypoxemia (PaO₂ < 50 mmHg), hours: 4.2; Duration of Hypoxemia (PaO₂ < 65 mmHg), hours: 21.55	Severe n = 40	55% in ECMO-group; 56% in non-ECMO-group
Rubega et al. [29] (MoCa values received after request to authors on 18 July 2024 (as raw data))	n = 16, age and male in % not described in MoCa group	Not described in MoCa group	Not described in MoCa group	MoCa	12 to 14 months after ICU discharge	Not described	Not described	Not described	62.5
Sturgill et al. [20]	n = 94, mean age: 52.72 (± 14.91), 51.1% male	M (SD): 16.6 (± 12.03)	M (SD): 22.47 (14.51)	MoCa	1–3 months after hospital discharge	n = 13	Not described	Not described	42.4
Vanginderhuysen et al. [30]	n = 45, mean age: 63.0 (± 11), 64.0% male	Not described in RBANDS/TMT group	Not described in RBANDS/TMT group	RBANDS/TMT	M (SD): 180	Not described	Not described	Not described	33
Duindam et al. [31]	n = 96, median age: 61 (55–69), 66.7% male	Median (IQR): 13 (6–23)	Median (IQR): 15 [18–28]	MoCa	M (SD): 6.5 (1.3)	Not described	136 mmHg	Mild n = 18; Moderate n = 54; Severe n = 24	5.2
Jaquet et al. [32]	n = 33, age and male in % not described in MoCA group	Not described in MoCA group	Not described in MoCA group	MoCa	3.8 (3.6–5.9) months	Not described in MoCA group	Not described in MoCA group	Not described in MoCA group	52
Latronico et al. [33]	n = 98, age median: 60 (52–66), 77% male	M (SD): 10.0 (± 8.0)	Median (IQR): 12 [7–21]	MoCa	3 months	Not described in MoCA group	Not described in MoCA group	Not described in MoCA group	28

For the meta-analyses, data from 14 studies including 650 participants with ARDS (of any cause) were analyzed. Within the subgroup of COVID-19-associated ARDS (C-ARDS), 12 studies comprising 563 participants were included.The samples vary in size, ranging from 13 to 98 participants. The average age falls between 50 and 70 years, with the proportion of male participants ranging from 48.3 to 90%. Some studies, however, do not report age or gender data for specific groups. The duration of mechanical ventilation spans from a few days (e.g., a median of 6 days in Okazaki et al., 2022) to 37 days (median in Martinez et al., 2023) [22, 25]. Similarly, the ICU length of stay varies ranges from 8 days (Okazaki et al., 2022) to 43 days (Silvestre et al., 2019) [22, 28]. Only Okazaki et al. described incidences in the cohort [22] while all other studies reported prevalence rates. Other important information that has a decisive influence on the cognitive performance of ARDS survivors—such as ARDS severity, the PaO₂/FiO₂ ratio, and ECMO use—was frequently included in the studies, but not always clearly in relation to the subgroup that underwent cognitive assessment.

Various standardized cognitive assessments were employed across the studies, including MoCA, MoCA-Blind, WAIS-IV, and RBANS/TMT. The choice of the outcome measurement instrument depended on the study and target population, with MoCA being the most commonly used (k = 10). The intervals between ICU discharge and cognitive assessment also varied. Some studies conducted assessments immediately after discharge (e.g., Martinez et al., 2023), while others waited several months (e.g., Latronico et al.,2022 at 3 months, Sylvestre et al., 2019 up to 21 months) [28, 33]. Both Pozzi et al. (2023) and Latronico et al. (2022) included multiple assessment time points. For the meta-analysis, the time points closest to the typical post-discharge assessment interval were included, such as 3 months for Latronico et al. (2022) and 96 days for Pozzi et al. (2023) [23, 33].

### Risk of bias assessment

The assessments of the included studies ranged from 5 to 7 points, indicating an overall moderate to low risk of bias. Three studies scored 7 points, indicating a fair (or moderate) risk of bias (Silvestre et al., 2019; Vanginderhuysen et al., 2024; Duindam et al., 2022). These studies were characterised by robust sample selection, good comparability between groups and reliable collection of results. The majority of studies (e.g. Okazaki et al., 2022; Pozzi et al., 2023; Maley et al., 2022; Martinez et al., 2023) scored 6 points. Some studies (Beaud et al., 2021; Monti et al., 2021; Weidman et al., 2022; Sturgill et al., 2023) only scored 5 points. Details of the assessments can be found in SC 5.

### Overall and subgroup analyses

**Fig. 2:**
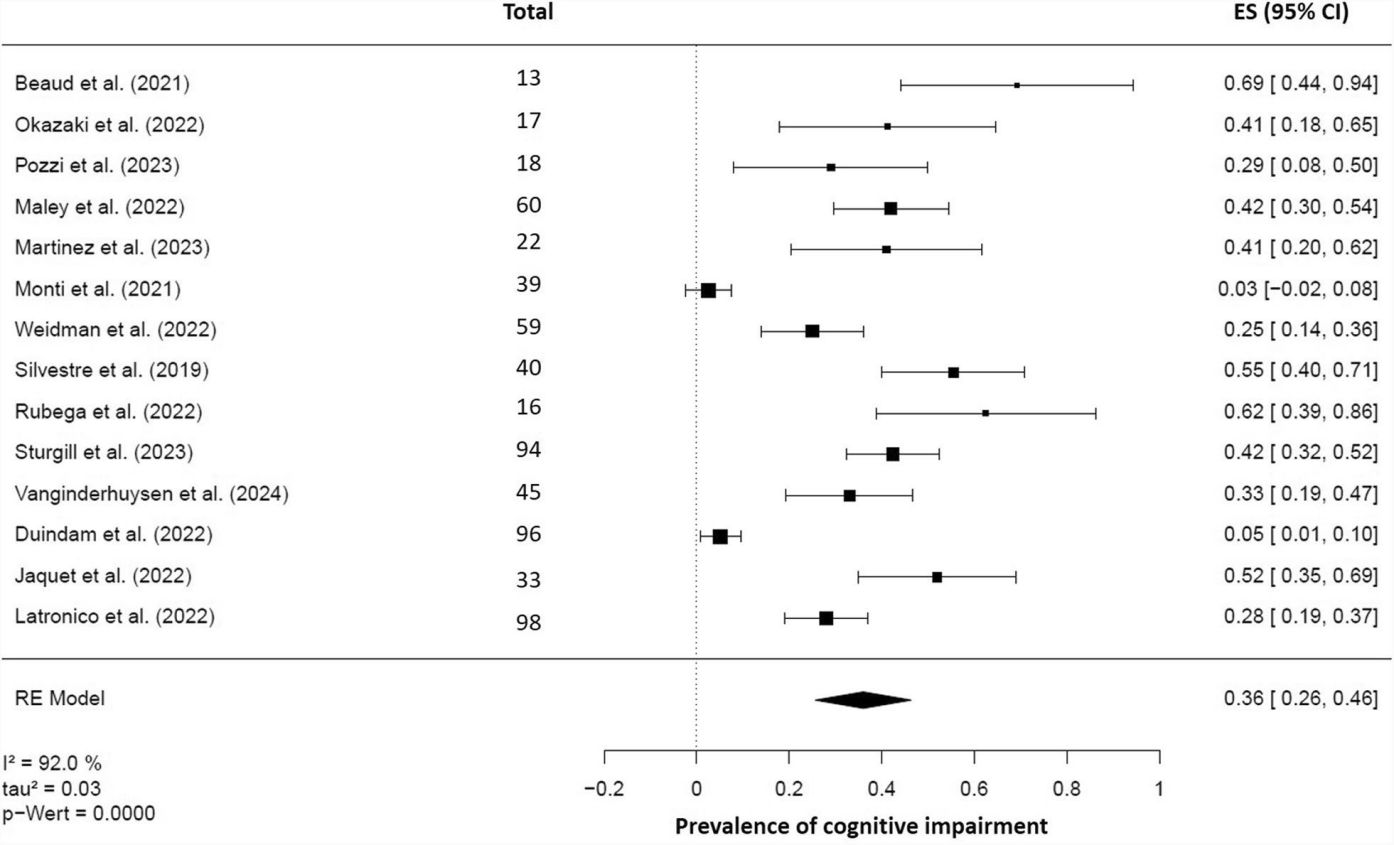
Forest plot resulting from random-effects meta-analyses of cognitive impairment following ARDS of any cause

The meta-analysis of the included studies (k = 14) revealed a prevalence rate of 36% (95% CI 26–46%, *p* < 0.001) for cognitive impairment after ARDS (Fig. 2). However, heterogeneity between studies was high (I^2^ = 92.0%, τ^2^ = 0.03).

**Fig. 3:**
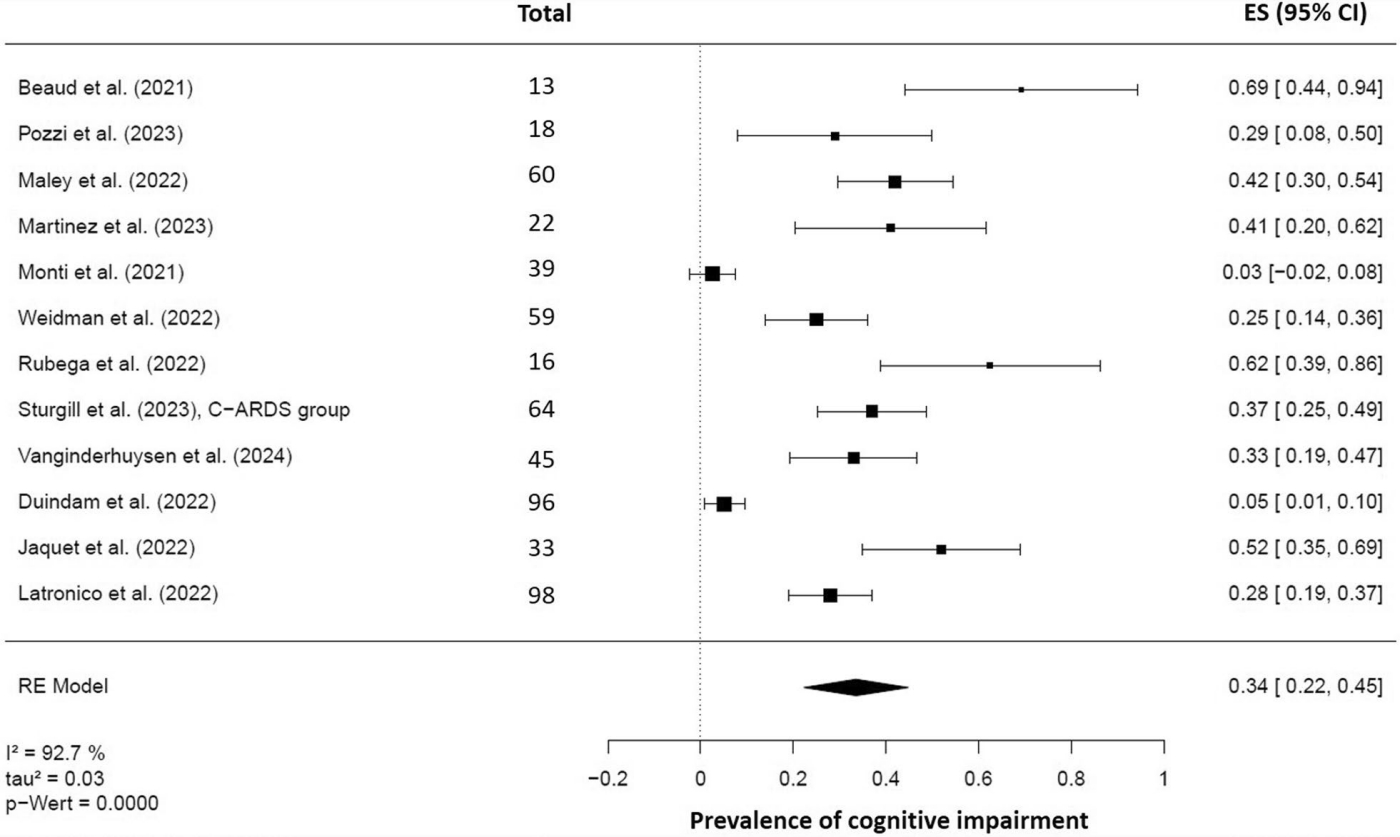
Forest plot resulting from random-effects meta-analyses of cognitive impairment following C-ARDS

When considering only the C-ARDS cohorts (k = 12), the pooled prevalence rate was 34% (95% CI 22–45%, *p* < 0.001), which is almost identical to the overall prevalence, while also showing high heterogeneity (I^2^ = 92.7%, τ^2^ = 0.03) (Fig. 3). The funnel plot and Egger's test suggested potential publication bias (SC 6). A subgroup analysis that only included studies with MoCA-based prevalences (k = 10) yielded a pooled prevalence rate of 37.7% (95% CI 26–49%, *p* < 0.001). Despite this restriction, there was a high degree of heterogeneity (I^2^ = 89.56%, τ^2^ = 0.03).

Multiple meta-regression showed that age was a significant predictor, with older age being associated with a higher prevalence of cognitive impairment after ARDS (b = 0.02, p = 0.033), reducing between study heterogeneity by roughly 35% (I^2^ = 60.04%, τ^2^ = 0.01). ICU stay (b: 0.02, p = 0.130 ), sex (b: 0.00, p = 0.195), and time between ICU discharge and cognitive assessment (b: 0.00, p = 0.221) did not show significant associations. In the Supplement, we provide both meta-regression scatter plots (SC 7) and bubble plots (SC 8) for regression models using one predictor at a time to visualize associations.

### Certainty of evidence assessments

The evidence is uncertain about the effect on the prevalence of cognitive impairment following both ARDS and C-ARDS, as the certainty of the findings was low due to the methodological limitations and high risk of bias in the included studies. This assessment is based on 14 and 12 studies, respectively, which provided observational data. The downgrade in evidence certainty for both subgroups was driven by significant methodological limitations, substantial heterogeneity, and indirectness (Table 3).

**Table 3:** Certainty of evidence assessment

Certainty assessments							Certainty
No of studies	Study design	Risk of bias	Inconsistency	Indirectness	Imprecision	Other considerations	
*Prevalence and incidence of cognitive impairment following ARDS of any cause*							
14	Observational studies	Serious^a^	Very serious^b^	Serious^c^	Not serious^d^	The funnel plot showed slight asymmetry, suggesting potential publication bias. Meta-regression analyses found no significant effects of predictors, with high heterogeneity across models. The results are not strongly influenced by test methods or study selection	++◯◯ low
*Prevalence and incidence of cognitive impairment following C-ARDS*							
12	Observational studies	Serious^e^	Very serious^f^	Serious^c^	Not serious^d^	The funnel plot showed slight asymmetry, suggesting potential publication bias. Meta-regression analyses found no significant effects of predictors, with high heterogeneity across models. The results are not strongly influenced by test methods or study selection	++◯◯ low

^a^Four out of 14 studies are classified as "Poor," indicating significant methodological weaknesses according to the NOS. These studies carry a high risk of bias, which could negatively impact the results of the analysis. The remaining ten studies are rated as "Fair," suggesting some weaknesses, but not necessarily critical ones

^b^I^2^ statistics indicate high heterogeneity (92.0%)

^c^These are exclusively adult ICU cohorts, which are relatively similar in terms of age distribution and gender ratio. However, there are significant differences in region, primary diagnosis, treatment duration, and other factors that were not clearly described (e.g., comorbidities)

^d^The 95% confidence interval is narrow, and the sample size in most studies is high

^e^Four out of 12 studies are classified as "Poor," indicating significant methodological weaknesses according to the NOS. These studies carry a high risk of bias, which could negatively impact the results of the analysis. The remaining ten studies are rated as "Fair," suggesting some weaknesses, but not necessarily critical ones

^f^I^2^ statistics indicate high heterogeneity (92.7%)

## Discussion

The objective of this systematic review and meta-analysis was to estimate the overall prevalence of cognitive impairments following ARDS, regardless of aetiology. A total of 14 studies involving 1451 adult ARDS survivors were identified, incorporating data published from 2013 through the COVID-19 pandemic. The pooled prevalence of cognitive impairments was estimated to be 36% (95% CI 26–46%) for all ARDS cases, with low certainty, and 34% (95% CI 22–45%) for C-ARDS specifically, also with low certainty. However, the evidence remains uncertain about the effect due to the methodological limitations of the included studies, which were characterized by a high risk of bias.

Although we were able to present the prevalence of cognitive impairments following ARDS, which was the primary goal of our study, it is important to note that we did not include, nor could we include, all key predictors that may contribute to the development of cognitive impairments after ARDS in our meta-analysis. In particular, predictors such as the PaO_2_/FiO_2_ ratio, use of ECMO, and the severity of ARDS are known to significantly impact cognitive performance. However, in the studies analyzed, these factors were not sufficiently recorded for the subpopulation that underwent cognitive assessment, limiting our ability to investigate their specific contributions.

Among these predictors, the severity of ARDS is directly correlated with the extent of cognitive impairments in survivors. Research indicates that up to 78% of patients with severe ARDS experience deficits in memory, attention, concentration, and mental processing speed [35]. These impairments can persist for years, significantly reducing the quality of life of ARDS survivors [34]. One of the key mechanisms underlying this association may be prolonged hypoxemia, systemic inflammation, and critical illness-related encephalopathy, all of which are more pronounced in severe ARDS cases.

Further supporting this association, Marsh et al. (2021) investigated the cognitive and psychological outcomes of patients with severe hypoxemia treated with ECMO, comparing them to a healthy control group [36]. While over 50% of patients reported cognitive difficulties (e.g., memory, attention, language), formal neuropsychological tests did not reveal significant impairments at the group level**.** However, a subset of ECMO patients exhibited deficits, particularly in executive function (4.2–8.3%) and working memory (4.2%)**,** with severe anterograde memory impairments found in 16.7% and significant overall memory deficits in 29.2% of patients. These findings highlight the complexity of post-ARDS cognitive impairment, suggesting that subjective cognitive complaints may not always align with formal assessments**,** and that some individuals remain at high risk despite seemingly normal group-level outcomes [36].

Due to the strong interrelation between PaO₂/FiO₂ ratio, ARDS severity, and the use of ECMO**,** these predictors may exhibit multicollinearity in further meta-regressions, making it difficult to determine their independent effects. A similar pattern was observed in our analysis, where mechanical ventilation and ICU stay showed high collinearity, ultimately necessitating the exclusion of mechanical ventilation as a predictor.

The pathophysiology of cognitive impairments in critically ill patients remains multifactorial [10], complicating the direct attribution of ARDS to cognitive impairment. Additionally, well-established predictors of cognitive impairments post-ARDS, such as hypotension, venous pressure, hyperglycemia, and amnesia at ICU admission or the duration of delirium [34, 37, 38], were seldom documented in the studies and could thus not be considered in our analysis. With the exception of the predictor age, sociodemographic and clinical factors were not associated with cognitive function after ARDS according to our meta-regressions. However, previous studies have identified older age as a relevant risk factor for cognitive impairment or delirium following intensive care. Meta-analyses, for example, reported a significantly increased risk of postoperative delirium with each additional year of age (OR = 1.06), as well as for patients over 65 years (OR = 3.21) [39].

Honarmand et al. (2020) quantified the prevalence of cognitive impairment among ICU survivors based on the type of cognitive testing methods used and the underlying condition [12]. Subjective assessments showed a prevalence of 35% [95% CI 29–41%] three months after discharge, which closely aligns with our findings of 36% [95% CI 26–46%]. However, objective tests resulted in higher prevalence rates (54% [95% CI 51–57%]) [12]. In our study, subjective methods, particularly the MoCA, were predominantly used, yielding a prevalence of 37.7% (95% CI 26–49%), which is consistent with the main outcome. Nevertheless, these figures may be less precise compared to objective measurements (e.g., neuropsychological test batteries).

The complexity of cognitive outcomes post-ARDS becomes even more evident when considering the syndrome's heterogeneity. The development of cognitive impairment after ARDS is influenced by the highly heterogeneous aetiology and varying severity of the syndrome [10]. Risk factors present before intensive care treatment are also associated with the subsequent recovery of ARDS survivors, particularly concerning their quality of life related to mental health [40]. However, only seven studies provided detailed data on the definition and severity of ARDS according to the Berlin definition [25, 22, 26, 28, 31–33]. Documented differences in the severity and course of ARDS could further contribute to the variability in prevalence rates. Sasannejad et al. (2019) emphasize that the interplay of clinical risk factors and pathophysiological events plays a critical role. Complete recovery from consequences, including cognitive impairment, does not always occur, and symptoms can persist for up to five years post-ICU discharge [10]. The systematic review by Wolters et al. (2013) identified a broad spectrum of cognitive impairment, with prevalence rates ranging from 4 to 62% across follow-up periods of 2 to 156 months, and concluded that the incidence of cognitive impairments is high following ICU discharge but tends to improve within the first few months post-discharge [11]. Wilcox et al. (2013) reported that the prevalence of cognitive impairment among ARDS survivors was between 70 and 100% at discharge, decreasing to 46–78% after one year, 25–47% after two years, and 20% after five years [9]. Honarmand et al. (2020), on the other hand, observed an increase in the prevalence of cognitive impairment, rising from 35% at three months post-discharge to 45% after more than six months [12]. Our analysis included two primary studies with multiple follow-up points [33, 23]. While Latrinoco et al. (2022) reported a decrease in prevalence from 28% at three months to 16% at twelve months [33]. Pozzi et al. (2023) showed an increase from 29 to 36% over the same period [23]. Other long-term studies also highlight the persistent morbidity effects of ARDS, even independently of cognitive vitality [41].

Many of the primary studies excluded patients with pre-existing cognitive impairments or neurodegenerative diseases, and none of the studies provided data on cognitive function prior to the ARDS event, likely due to the acute emergency situation of an ICU-bound ARDS incident. However, this is particularly relevant, as pre-existing cognitive impairment is considered a risk factor for further cognitive decline after a critical illness [42]. This absence of baseline data underscores a significant methodological limitation.

Furthermore, in our meta-analysis, it was important to distinguish between C-ARDS and typical ARDS, as C-ARDS is mainly characterized by its early stages differing from classic ARDS in severe pneumonia and is associated with different ventilation strategies [43]. Given that up to 40% of patients hospitalized with COVID-19 pneumonia develop ARDS [44, 45], it is not surprising that a significant proportion of primary studies have focused on C-ARDS in recent years.

MCI, recognized as a transitional phase toward Alzheimer's disease and other neurodegenerative conditions [46] further emphasizes the need for this meta-analysis to assess cognitive outcomes in ARDS survivors. Globally, the overall prevalence of MCI is 15.56% among adults aged 50 years and older [47], highlighting the widespread nature of this condition Despite the increase in both the number of insured individuals diagnosed with MCI—from 51,000 in 2009 to 198,000 in 2018 in Germany—and the rising number of dementia cases, there remains a significant gap between diagnosed MCI cases and the estimated actual number of MCI patients**,** suggesting a likely high rate of underdiagnosis and missed early detection opportunities. As the mortality rate among ARDS survivors continues to decrease over time [48], and considering that cognitive impairment may not initially disrupt daily life activities but can significantly increase the risk of dementia later in life, the importance of further research into this topic is underscored [49, 50].

### Limitations and strengths of the current review

Our findings are influenced by several limitations of the included studies. One of the most critical issues is that only two primary studies provided data on cognitive impairment in patients without C-ARDS. The evidence is uncertain about the effect, as the available data for this subgroup is of very low certainty, making it difficult to draw reliable conclusions regarding the prevalence or incidence of cognitive impairment in these patients.

One major limitation of this study is the very high heterogeneity in the meta-analysis, which strongly limits the interpretability of the results. The substantial variability across studies, stemming from differences in study designs, cognitive assessment methods, follow-up durations, and patient characteristics, makes it challenging to draw definitive conclusions about the true prevalence and predictors of cognitive impairment after ARDS.

A potential small-study effect cannot be ruled out in our meta-analysis. Egger’s regression test and the funnel plot analysis indicated signs of publication bias, suggesting that smaller studies might have reported disproportionately larger effect sizes. This could be due to the selective publication of significant findings, methodological limitations in smaller studies, or random variance in effect estimates. While small-study effects are a common challenge in meta-analyses, their presence in our study highlights the need for cautious interpretation of the results.

While our meta-analysis included 14 studies meeting our eligibility criteria, we observed substantial variability in the availability of relevant data within these studies. In several cases, ARDS patients constituted only a subset of the total study population, and cognitive outcomes were assessed in only a fraction of these ARDS patients. Consequently, key clinical parameters such as PaO₂/FiO₂ ratio, ECMO use, and prone positioning, although reported in some studies, were often not available for the specific subgroup of interest. This discrepancy should be considered when interpreting our findings, as it may introduce selection bias and affect the generalizability of the results.

Additionally, the follow-up periods in the studies vary significantly (ranging from immediate post-discharge assessments to up to one year later) and data on long-term developments is lacking, which complicates the comparison of results and prevents a full understanding of the temporal progression of cognitive impairment.

Despite investigating potential predictors of cognitive impairment through meta-regressions, no significant associations were identified. This may reflect insufficient data or statistical power to detect an effect, or the complexity of multifactorial relationships. Further, some relevant studies could not be included as authors did not provide raw data.

Although there are these shortcomings, the current meta-analysis provides valuable insights into the prevalence of cognitive impairment following ARDS and C-ARDS. Our analysis is based on a comprehensive literature search across three major databases, supplemented by a review of reference lists and citations from relevant studies. Additionally, the use of established tools for RoB and GRADE assessments strengthens the reliability of our findings. Furthermore, reporting according to PRISMA and MOOSE guidelines, along with key steps being carried out by at least two independent reviewers, ensures the methodological rigor of this analysis.

## Conclusions

This meta-analysis indicates that cognitive impairment is a common long-term consequence, although the certainty of the evidence is low. While post-COVID-19 cognitive impairment has received significant attention, our findings suggest that these persistent symptoms are not unique to COVID-19 but rather part of the broader spectrum of ARDS-related cognitive impairments. The consistent prevalence of cognitive deficits in our analysis underscores that ARDS itself poses a long-term risk to cognitive function, irrespective of its etiology. Future research should not only combine objective and subjective cognitive tests but should also utilize longitudinal designs to better capture changes in cognitive function over time. Additionally, it would be important to integrate both pre- and post-morbid cognitive data to enable a more nuanced analysis of ARDS-related effects. By doing so, a clearer understanding of the long-term cognitive consequences of ARDS could be achieved, informing therapeutic interventions and supporting strategies for affected patients.

## Supplementary Information

Additional file 1.

## Data Availability

No datasets were generated or analysed during the current study.

## Abbreviations

ARDS: Acute respiratory distress syndrome
C-ARDS: COVID-19-associated ARDS
GRADE: Grading of Recommendations, Assessment, Development and Evaluations
ICU: Intensive care unit
MCI: Mild cognitive impairment
MoCA: Montreal Cognitive Assessment
MOOSE: Meta-analysis of Observational Studies in Epidemiology
NOS: Newcastle-Ottawa Scale
PRISMA: Preferred Reporting Items for Systematic Reviews
REM: Random-effects model
REML: Restricted maximum likelihood (REML)
SC: Supplemental Content
WAIS-IV: Wechsler Adult Intelligence Scale, 4th edition
