# Supplementary material for: Prevalence and incidence of cognitive impairment following acute respiratory distress syndrome of any cause: a systematic review and meta-analysis
Source: Crit Care. 2025 Apr 23;29:164. doi: 10.1186/s13054-025-05375-x (PMC12020237; doi:10.1186/s13054-025-05375-x)
Supplement: Supplementary file 1 — Additional file 1. [file 13054_2025_5375_MOESM1_ESM.docx]

**Supplemental Content - Prevalence and incidence of cognitive impairment following acute respiratory distress syndrome of any cause: A systematic review and meta-analysis**

Supplemental Content (SC):

- SC 1 Full search strategy (page 2)
- SC 2 Guidelines with decision criteria for title/abstract screening (page 3-4)
- SC 3 Guidelines with decision criteria for full-text screening (page 5-7)
- SC 4 Fulltext screening: Excluded studies and reasons for exclusion (page 8-14)
- SC 5 Assessment by Newcastle-Ottawa Scale (page 15)
- SC 6 Funnel plots, publication bias (page 16-17)
- SC 7 Meta-regression scatter plots (page 18)
- SC 8 Bubble plots (page 20-21)

**SC 1**

**Full search strategy**

**PubMed**

("acute respiratory distress syndrome"[Title/Abstract] OR ARDS[Title/Abstract] OR "acute respiratory insufficiency syndrome"[Title/Abstract] OR "acute hypoxic respiratory failure"[Title/Abstract] OR "acute respiratory failure"[Title/Abstract] OR "acute lung injury"[Title/Abstract] OR ALI[Title/Abstract] OR "shock lung"[Title/Abstract] OR "shock lungs"[Title/Abstract]) OR ("post-intensive care syndrome"[Title/Abstract] OR PICS[Title/Abstract] OR "Intensive care"[title/abstract] OR "Intensive care unit"[title/abstract] OR ICU[title/abstract]) OR (COVID-19[title/abstract] OR "Severe Acute Respiratory Syndrome Coronavirus 2"[Title/Abstract] OR SARS-CoV-2[Title/Abstract]) AND ("cognitive impairment"[Title/Abstract] OR "mild cognitive impairment"[Title/Abstract] OR MCI[Title/Abstract] OR cognition[Title/Abstract] OR cognitive[Title/Abstract] OR cognitive dysfunction*[Title/Abstract] OR cognitive function*[Title/Abstract] OR neurologic*[Title/Abstract] OR "neuropsychological impairment"[Title/Abstract] OR "subjective cognitive decline"[Title/Abstract] OR Long-term[Title/Abstract]) AND (Prevalence[Title/Abstract] OR incidence[Title/Abstract] OR frequency[Title/Abstract] OR epidemiology[Title/Abstract])

**Scopus**

("acute respiratory distress syndrome" OR ards OR "acute respiratory insufficiency syndrome" OR "acute hypoxic respiratory failure" OR "acute respiratory failure" OR "acute lung injury" OR ali OR "shock lung" OR "shock lungs" ) OR ( "post-intensive care syndrome" OR pics OR "Intensive care" OR "Intensive care unit" OR icu ) OR ( covid-19 OR "Severe Acute Respiratory Syndrome Coronavirus 2" OR sars-cov-2 ) AND ( "cognitive impairment" OR "mild cognitive impairment" OR mci OR cognition OR cognitive OR "cognitive dysfunction*" OR "cognitive function*" OR neurologic* OR "neuropsychological impairment" OR "subjective cognitive decline" OR long-term ) AND ( prevalence OR incidence OR frequency OR epidemiology )

**Web of Scienes**

("acute respiratory distress syndrome" OR ARDS OR "acute respiratory insufficiency syndrome" OR "acute hypoxic respiratory failure" OR "acute respiratory failure" OR "acute lung injury" OR ALI OR "shock lung" OR "shock lungs" OR "post-intensive care syndrome" OR PICS OR "Intensive care" OR "Intensive care unit" OR ICU OR COVID-19 OR "Severe Acute Respiratory Syndrome Coronavirus 2" OR SARS-CoV-2) AND ("cognitive impairment" OR "mild cognitive impairment" OR MCI OR cognition OR cognitive OR "cognitive dysfunction*" OR "cognitive function*" OR neurologic* OR "neuropsychological impairment" OR "subjective cognitive decline" OR Long-term) AND (Prevalence OR incidence OR frequency OR epidemiology)

**SC 2**

**Guidelines with decision criteria for title/abstract screening**

**Guidelines with decision criteria for title/abstract screening**

"Prevalence and incidence of cognitive impairment following acute respiratory distress syndrome of any cause. A systematic review and meta-analysis"

_________________________________________________________________________________

**Decision criteria:**

These criteria apply to the entire review. The criteria remain the same throughout the entire review process.

**Outcome" decision criteria**

| **Inclusion criteria** | **Exclusion criteria** |
| --- | --- |
| - Observed or recorded event rate of cognitive impairment - Observed or recorded event rate of all synonyms for cognitive impairments (cognitive deficits, memory disorders, thinking problems, mental disorders, mental limitations, mental impairments, cognitive dysfunctions, thinking disorders)   - Delirium/delirium, as many authors do not distinguish between cognitive impairment and acute delirium - Event rate of cognitive impairment as a result of COVID-19, ICU or PICS (if ARDS is reported) | - Neurodegenerative diseases (dementia, Alzheimer's, etc.) - (Long-term) consequences of COVID-19 without recording on cognitive impairments - PICS without detection of cognitive impairment - Observation of ICU patients without recording cognitive impairments - Studies investigating delirium or postoperative cognitive dysfunction (POCD) on admission to the intensive care unit |

**Decision criteria "Exposure"**

| **Inclusion criteria** | **Exclusion criteria** |
| --- | --- |
| - People who have survived ARDS regardless of the triggering cause (including COVID-19) - All synonyms for ARDS (including acute respiratory distress syndrome, acute respiratory failure, respiratory syndrome) - ARDS regardless of the severity of hypoxaemia (mild, moderate, severe) - ARDS with and without mechanical ventilation - ARDS as a subcategory in PICS cohorts or ICU patients | - Studies that report on PICS but do not describe ARDS - Studies that report COVID-19 infection but do not describe ARDS |

**Population" decision criteria**

| **Inclusion criteria** | **Exclusion criteria** |
| --- | --- |
| - Adult patients (≥ 18 years) who have survived ARDS and have been diagnosed/observed with cognitive impairment | - Children and paediatric studies - Patients in whom traumatic brain injury and stroke have been reported - Patients after chemotherapy or during chemotherapy (chemobrain) |

**Decision criteria "Study design"**

| **Inclusion criteria** | **Exclusion criteria** |
| --- | --- |
| - Epidemiological observational studies (both prospective and retrospective) - Intervention studies (including randomised controlled trials, controlled clinical trials and before-and-after evaluations) - Cross-sectional and longitudinal studies - Prevalence studies | - Experimental and quasi-experimental studies - Qualitative studies (interviews, focus groups) - Genetic tests / genetic / blood tests - Systematic reviews (and meta-analyses) - Rapid reviews, scoping reviews, narrative reviews |

**Decision criteria "Publication form"**

| **Inclusion criteria** | **Exclusion criteria** |
| --- | --- |
| - Studies with data collection from 01.01.2013 in English or German - Publication form: - Peer review | - Preprints - Books, book chapters, book reviews - Editorial, commentary, expert opinion, corrections, letters to the editor, statements - Study protocols, guidelines, treatment protocols etc. - Introduction, Forewords - Popular science media - Only abstract available   e.g. workshops/ conference contributions   - Dissertations |

**SC 3**

**Guidelines with decision criteria for fulltext screening**

**Guidelines with decision criteria for full text review**

"Prevalence and incidence of cognitive impairment following acute respiratory distress syndrome of any cause. A systematic review and meta-analysis"

**General note for full text:**

- If a full text is excluded, the **reason for the exclusion** must be noted.
- If a full text appears to be relevant, even if a decision criterion does not apply, the study should then be included in the **decision uncertain** category and subsequently discussed in the group.
- The following **thematic prioritisation** should be applied when selecting the reason for exclusion:

| **Hierarchy level** | **Reason for exclusion** |
| --- | --- |
| 0 | Publication form not applicable |
| 1 | Study design |
| 2 | Exposure not applicable (or ARDS not clearly described) |
| 3 | Population not applicable |
| 4 | Outcome not applicable |
| 5 | Other (e.g. data collection before 2013 or not in English ch or German) |

🡪 If a full text is available in English or German, but the study design or publication form does not apply (e.g. it is an editorial, commentary, case report, interviews, etc.), "**Study design/publication form not applicable**" is given as the reason for exclusion.

🡪 If a full text is available in English or German, the study design is appropriate, but the exposure does not apply (patients after ARDS incident of any kind), "**Exposure not applicable**" is given as the reason for exclusion

🡪 If a full text is available in English or German, the study design and exposure are appropriate, but the population does not apply (e.g. paediatric cohorts with children or adolescents), "**Population not applicable**" is given as the reason for exclusion

🡪 If a full text is available in English or German, the study design, exposure and population are appropriate, but the required outcome (cognitive impairment of any kind) does not fit (e.g. neurodegenerative diseases are reported), the reason for exclusion is "**Outcome not applicable**".

🡪 If there is a completely different reason for exclusion not mentioned here, "Other" should be selected and the reason documented in writing (e.g. no thematic reference)

**Decision criteria:**

These criteria apply to the entire review. The criteria remain the same throughout the entire review process.

**decision criteria "Outcome"**

| **Inclusion criteria** | **Exclusion criteria** |
| --- | --- |
| - Observed or recorded event rate of cognitive impairment - Observed or recorded event rate of all synonyms for cognitive impairments (cognitive deficits, memory disorders, thinking problems, mental disorders, mental limitations, mental impairments, cognitive dysfunctions, thinking disorders) - Event rate of cognitive impairment as a result of COVID-19, ICU or PICS (**if ARDS is clearly reported**) | - Neurodegenerative diseases (dementia, Alzheimer's, etc.) - (Long-term) consequences of COVID-19 without recording on cognitive impairments - PICS without detection of cognitive impairment - Observation of ICU patients without recording cognitive impairments - Studies investigating only delirium or postoperative cognitive dysfunction (POCD) on admission to the ICU - Delirium/Delirium |

**Decision criteria "Exposure"**

| **Inclusion criteria** | **Exclusion criteria** |
| --- | --- |
| - People who have survived ARDS regardless of the triggering cause (including COVID-19) - All synonyms for ARDS (including acute respiratory distress syndrome, acute respiratory failure, respiratory syndrome) - ARDS regardless of the severity of hypoxaemia (mild, moderate, severe) - ARDS with and without mechanical ventilation - ARDS as a subcategory in PICS cohorts or ICU patients | - Studies that report on PICS but do not describe ARDS - Studies that report COVID-19 infection but do not describe ARDS |

**Population" decision criteria**

| **Inclusion criteria** | **Exclusion criteria** |
| --- | --- |
| - Adult patients (≥ 18 years) who have survived ARDS and have been diagnosed/observed with cognitive impairment | - Children and paediatric studies - Patients in whom traumatic brain injury and stroke have been reported - Patients after chemotherapy or during chemotherapy (chemobrain) |

**Decision criteria "Study design"**

| **Inclusion criteria** | **Exclusion criteria** |
| --- | --- |
| - Epidemiological observational studies (both prospective and retrospective) - Intervention studies (including randomised controlled trials, controlled clinical trials and before-and-after evaluations) - Cross-sectional and longitudinal studies - Prevalence studies | - Experimental and quasi-experimental studies - Qualitative studies (interviews, focus groups) - Genetic tests / genetic / blood tests - Systematic reviews (and meta-analyses) - Rapid reviews, scoping reviews, narrative reviews |

**Decision criteria "Publication form"**

| **Inclusion criteria** | **Exclusion criteria** |
| --- | --- |
| - Studies with data collection from 01.01.2013 in English or German - Cohorts from 01.01.2013 - Publication form: - Peer review | - Preprints - Books, book chapters, book reviews - Editorial, commentary, expert opinion, corrections, letters to the editor, statements - Study protocols, guidelines, treatment protocols etc. - Introduction, Forewords - Popular science media - Only abstract available   e.g. workshops/ conference contributions   - Dissertations |

**SC 4**

**Fulltext screening: Excluded studies and reasons for exclusion**

| **#** | **Study** | **Reason for exclusion** |
| --- | --- | --- |
|  | Neufeld KJ, Leoutsakos J-MS, Yan H, et al. Fatigue Symptoms During the First Year Following ARDS. *Chest*. 2020;158(3):999-1007. doi:10.1016/j.chest.2020.03.059 | 2 |
|  | Nanwani-Nanwani K, López-Pérez L, Giménez-Esparza C, et al. Prevalence of post-intensive care syndrome in mechanically ventilated patients with COVID-19. *Sci Rep*. 2022;12(1):7977. doi:10.1038/s41598-022-11929-8 | 5 |
|  | Nelliot A, Dinglas VD, O’Toole J, et al. Acute Respiratory Failure Survivors’ Physical, Cognitive, and Mental Health Outcomes: Quantitative Measures versus Semistructured Interviews. *Ann Am Thorac Soc*. 2019;16(6):731-737. doi:10.1513/AnnalsATS.201812-851OC | 1 |
|  | Needham DM, Colantuoni E, Dinglas VD, et al. Rosuvastatin versus placebo for delirium in intensive care and subsequent cognitive impairment in patients with sepsis-associated acute respiratory distress syndrome: an ancillary study to a randomised controlled trial. *Lancet Respir Med*. 2016;4(3):203-212. doi:10.1016/S2213-2600(16)00005-9 | 5 |
|  | Needham DM, Dinglas VD, Bienvenu OJ, et al. One year outcomes in patients with acute lung injury randomised to initial trophic or full enteral feeding: prospective follow-up of EDEN randomised trial. *BMJ*. 2013;346:f1532. doi:10.1136/bmj.f1532 | 5 |
|  | Needham DM, Dinglas VD, Morris PE, et al. Physical and cognitive performance of patients with acute lung injury 1 year after initial trophic versus full enteral feeding. EDEN trial follow-up. *Am J Respir Crit Care Med*. 2013;188(5):567-576. doi:10.1164/rccm.201304-0651OC | 5 |
|  | Oh TK, Park HY, Song I-A. Factors associated with delirium among survivors of acute respiratory distress syndrome: a nationwide cohort study. *BMC Pulm Med*. 2021;21(1):341. doi:10.1186/s12890-021-01714-0 | 4 |
|  | Ofoma UR, Reriani MK, Biehl M, et al. Neuromuscular and neuropsychological assessments in survivors of acute respiratory distress syndrome: exploratory comparisons with an at-risk cohort. *Neurocrit Care*. 2015;22(3):395-402. doi:10.1007/s12028-014-0087-4 | 4 |
|  | Norman BC, Jackson JC, Graves JA, et al. Employment Outcomes After Critical Illness: An Analysis of the Bringing to Light the Risk Factors and Incidence of Neuropsychological Dysfunction in ICU Survivors Cohort. *Crit Care Med*. 2016;44(11):2003-2009. doi:10.1097/CCM.0000000000001849 | 2, 5 |
|  | Peghin M, Palese A, Venturini M, et al. Post-COVID-19 symptoms 6 months after acute infection among hospitalized and non-hospitalized patients. *Clin Microbiol Infect*. 2021;27(10):1507-1513. doi:10.1016/j.cmi.2021.05.033 | 1, 4 |
|  | Roedl K, Söffker G, Fischer D, et al. Effects of COVID-19 on in-hospital cardiac arrest: incidence, causes, and outcome - a retrospective cohort study. *Scand J Trauma Resusc Emerg Med*. 2021;29(1):30. doi:10.1186/s13049-021-00846-w | 4 |
|  | Pfoh ER, Chan KS, Dinglas VD, et al. Cognitive screening among acute respiratory failure survivors: a cross-sectional evaluation of the Mini-Mental State Examination. *Crit Care*. 2015;19(1):220. doi:10.1186/s13054-015-0934-5 | 5 |
|  | Piva S, Pozzi M, Bellani G, et al. Long-term physical impairments in survivors of COVID-19-associated ARDS compared with classic ARDS: A two-center study. *J Crit Care*. 2023;76:154285. doi:10.1016/j.jcrc.2023.154285 | 4 |
|  | Probert JM, Lin S, Yan H, et al. Bodily pain in survivors of acute respiratory distress syndrome: A 1-year longitudinal follow-up study. *J Psychosom Res*. 2021;144:110418. doi:10.1016/j.jpsychores.2021.110418 | 5 |
|  | Martín-Jiménez P, Muñoz-García MI, Seoane D, et al. Cognitive Impairment Is a Common Comorbidity in Deceased COVID-19 Patients: A Hospital-Based Retrospective Cohort Study. *J Alzheimers Dis*. 2020;78(4):1367-1372. doi:10.3233/JAD-200937 | 3 |
|  | Marra A, Pandharipande PP, Girard TD, et al. Co-Occurrence of Post-Intensive Care Syndrome Problems Among 406 Survivors of Critical Illness. *Crit Care Med*. 2018;46(9):1393-1401. doi:10.1097/CCM.0000000000003218 | 2 |
|  | Marsh LC, Leach RM, Blane J, et al. Long-term cognitive and psychiatric outcomes of acute respiratory distress syndrome managed with Extracorporeal Membrane Oxygenation. *Respir Med*. 2021;183:106419. doi:10.1016/j.rmed.2021.106419 | 2 |
|  | Möller CM, Ellmauer P-P, Zeman F, et al. Postoperative acute respiratory dysfunction and the influence of antibiotics after acute type A aortic dissection surgery: A retrospective analysis. *PLoS One*. 2021;16(2):e0246724. doi:10.1371/journal.pone.0246724 | 4 |
|  | Mazeraud A, Robba C, Rebora P, et al. Acute Distress Respiratory Syndrome After Subarachnoid Hemorrhage: Incidence and Impact on the Outcome in a Large Multicenter, Retrospective Cohort. *Neurocrit Care*. 2021;34(3):1000-1008. doi:10.1007/s12028-020-01115-x | 4 |
|  | Mattioli F, Piva S, Stampatori C, et al. Neurologic and cognitive sequelae after SARS-CoV2 infection: Different impairment for ICU patients. *J Neurol Sci*. 2022;432:120061. doi:10.1016/j.jns.2021.120061 | 5 |
|  | Mitchell ML, Shum DHK, Mihala G, Murfield JE, Aitken LM. Long-term cognitive impairment and delirium in intensive care: A prospective cohort study. *Aust Crit Care*. 2018;31(4):204-211. doi:10.1016/j.aucc.2017.07.002 | 2 |
|  | Wang G, He S, Yu M, Zhang Y, Mu D, Wang D. Intraoperative body temperature and emergence delirium in elderly patients after non-cardiac surgery: A secondary analysis of a prospective observational study. *Chin Med J (Engl)*. 2023;136(19):2330-2339. doi:10.1097/CM9.0000000000002375 | 2 |
|  | Wang D, Hu B, Hu C, et al. Clinical Characteristics of 138 Hospitalized Patients With 2019 Novel Coronavirus-Infected Pneumonia in Wuhan, China. *JAMA*. 2020;323(11):1061-1069. doi:10.1001/jama.2020.1585 | 2, 4 |
|  | Wang X-P, Lv D, Chen Y-F, et al. Impact of Pain, Agitation, and Delirium Bundle on Delirium and Cognitive Function. *J Nurs Res*. 2022;30(4):e222. doi:10.1097/jnr.0000000000000497 | 2 |
|  | Wang Z-Y, Li T, Wang C-T, Xu L, Gao X-J. Assessment of 1-year Outcomes in Survivors of Severe Acute Respiratory Distress Syndrome Receiving Extracorporeal Membrane Oxygenation or Mechanical Ventilation: A Prospective Observational Study. *Chin Med J (Engl)*. 2017;130(10):1161-1168. doi:10.4103/0366-6999.205847 | 4 |
|  | Wardani, Dyah Wulan Sumekar Rengganis, Pramesona BA, Nirmala Y. Physical and psychological effects of post-COVID-19 syndrome on patients in Bandar Lampung, Indonesia: a descriptive prospective study. *Journal of Current Science and Technology*. 2023;13(2):317-325. doi:10.59796/jcst.V13N2.2023.1746 | 2 |
|  | Wassenaar A, van den Boogaard M, van Achterberg T, et al. Multinational development and validation of an early prediction model for delirium in ICU patients. *Intensive Care Med*. 2015;41(6):1048-1056. doi:10.1007/s00134-015-3777-2 | 2 |
|  | Watanabe S, Kotani T, Taito S, et al. Determinants of gait independence after mechanical ventilation in the intensive care unit: a Japanese multicenter retrospective exploratory cohort study. *J Intensive Care*. 2019;7:53. doi:10.1186/s40560-019-0404-2 | 2 |
|  | Umbrello M, Miori S, Sanna A, et al. High rates of impaired quality of life and social and economic problems at 6 months after COVID-19-related ARDS. *J Anesth Analg Crit Care*. 2022;2(1):20. doi:10.1186/s44158-022-00048-5 | 4 |
|  | Veeravagu A, Chen Y-R, Ludwig C, et al. Acute lung injury in patients with subarachnoid hemorrhage: a nationwide inpatient sample study. *World Neurosurg*. 2014;82(1-2):e235-41. doi:10.1016/j.wneu.2014.02.030 | 4 |
|  | Varón-Vega FA, Hernández-Parra Á, Molina F, et al. Epidemiology, clinical evolution and outcomes of tracheobronchitis and pneumonia associated with mechanical ventilation in intensive care units of Latin American; *Infectio*. 2017;21(2):74-80. doi:10.22354/in.v21i2.650 | 5 |
|  | Williamson CA, Faiver L, Nguyen AM, Ottenhoff L, Rajajee V. Incidence, Predictors and Outcomes of Delirium in Critically Ill Patients With COVID-19. *Neurohospitalist*. 2022;12(1):31-37. doi:10.1177/19418744211034815 | 2, 4 |
|  | Zhang HG, Dagliati A, Shakeri Hossein Abad Z, et al. International electronic health record-derived post-acute sequelae profiles of COVID-19 patients. *NPJ Digit Med*. 2022;5(1):81. doi:10.1038/s41746-022-00623-8 | 2 |
|  | Zhang S, Bai W, Yue J, et al. Eight months follow-up study on pulmonary function, lung radiographic, and related physiological characteristics in COVID-19 survivors. *Sci Rep*. 2021;11(1). doi:10.1038/s41598-021-93191-y | 2, 4 |
|  | Yuksel H, Gursoy GT, Dirik EB, et al. Neurological manifestations of COVID-19 in confirmed and probable cases: A descriptive study from a large tertiary care center. *J Clin Neurosci*. 2021;86:97-102. doi:10.1016/j.jocn.2021.01.002 | 2, 4 |
|  | Zhu W, Bai Y, Li S, et al. Delirium in hospitalized COVID-19 patients: a prospective, multicenter, cohort study. *J Neurol*. 2023;270(10):4608-4616. doi:10.1007/s00415-023-11882-0 | 2, 4 |
|  | Zhou P, Xu H, Li B, et al. Neurological outcomes in adult drowning patients in China. *Ann Saudi Med*. 2022;42(2):127-138. doi:10.5144/0256-4947.2022.127 | 2 |
|  | Zhang W, Wu W, Gu J, et al. Risk factors for postoperative delirium in patients after coronary artery bypass grafting: A prospective cohort study. *J Crit Care*. 2015;30(3):606-612. doi:10.1016/j.jcrc.2015.02.003 | 2 |
|  | Wong EK-C, Watt J, Zou H, et al. Characteristics, treatment and delirium incidence of older adults hospitalized with COVID-19: a multicentre retrospective cohort study. *CMAJ Open*. 2022;10(3):E692-E701. doi:10.9778/cmajo.20210176 | 2, 4 |
|  | Wilson ME, Barwise A, Heise KJ, et al. Long-Term Return to Functional Baseline After Mechanical Ventilation in the ICU. *Crit Care Med*. 2018;46(4):562-569. doi:10.1097/CCM.0000000000002927 | 2 |
|  | Wintermann G-B, Rosendahl J, Weidner K, Strauß B, Hinz A, Petrowski K. Self-reported fatigue following intensive care of chronically critically ill patients: a prospective cohort study. *J Intensive Care*. 2018;6:27. doi:10.1186/s40560-018-0295-7 | 4 |
|  | Wozniak AW, Pfoh ER, Dinglas VD, Pronovost PJ, Needham DM, Colantuoni E. Hospital Readmission and Subsequent Decline in Long-Term Survivors of Acute Respiratory Distress Syndrome. *Am J Crit Care*. 2019;28(1):76-80. doi:10.4037/ajcc2019580 | 4 |
|  | Yang L, Chen W, Chen D, et al. Cohort profile: the China surgery and anesthesia cohort (CSAC). *Eur J Epidemiol*. 2024. doi:10.1007/s10654-023-01083-4 | 0, 2 |
|  | Yao L, Li Y, Yin R, et al. Incidence and influencing factors of post-intensive care cognitive impairment. *Intensive Crit Care Nurs*. 2021;67:103106. doi:10.1016/j.iccn.2021.103106 | 2 |
|  | Ruhl AP, Huang M, Colantuoni E, et al. Healthcare utilization and costs in ARDS survivors: a 1-year longitudinal national US multicenter study. *Intensive Care Med*. 2017;43(7):980-991. doi:10.1007/s00134-017-4827-8 | 5 |
|  | Rousseau A-F, Minguet P, Colson C, et al. Post-intensive care syndrome after a critical COVID-19: cohort study from a Belgian follow-up clinic. *Ann Intensive Care*. 2021;11(1):118. doi:10.1186/s13613-021-00910-9 | 2 |
|  | Taniguchi LU, Aliberti MJR, Dias MB, Jacob-Filho W, Avelino-Silva TJ. Twelve Months and Counting: Following Clinical Outcomes in Critical COVID-19 Survivors. *Ann Am Thorac Soc*. 2023;20(2):289-295. doi:10.1513/AnnalsATS.202207-630OC | 4 |
|  | Szymczak H, Dodoo-Schittko F, Brandstetter S, et al. Trajectories of quality of life, return to work, psychopathology, and disability in survivors of the acute respiratory distress syndrome (ARDS): A three-year prospective cohort study (DACAPO). *J Crit Care*. 2023;78:154356. doi:10.1016/j.jcrc.2023.154356 | 4 |
|  | Tong C, Huang C, Wu J, Xu M, Cao H. The Prevalence and Impact of Undiagnosed Mild Cognitive Impairment in Elderly Patients Undergoing Thoracic Surgery: A Prospective Cohort Study. *J Cardiothorac Vasc Anesth*. 2020;34(9):2413-2418. doi:10.1053/j.jvca.2020.03.011 | 2 |
|  | Tomasi R, Klemm M, Hinske CL, et al. Impairment of Cognitive Function in Different Domains Early After Lung Transplantation. *J Clin Psychol Med Settings*. 2022;29(1):103-112. doi:10.1007/s10880-021-09787-z | 2 |
|  | Teeters DA, Moua T, Li G, et al. Mild Cognitive Impairment and Risk of Critical Illness. *Crit Care Med*. 2016;44(11):2045-2051. doi:10.1097/CCM.0000000000001842 | 2 |
|  | Tejero-Aranguren J, Moral Martin RG-D, Poyatos-Aguilera ME, Morales-Galindo I, Cobos-Vargas A, Colmenero M. Incidence and risk factors for postintensive care syndrome in a cohort of critically ill patients. *Rev Bras Ter Intensiva*. 2022;34(3):380-385. doi:10.5935/0103-507X.20220224-en | 2 |
|  | Tawakul AA, Alharbi AH, Basahal AM, et al. Neurological Symptoms and Complications of COVID-19 Among Patients in a Tertiary Hospital in Saudi Arabia. *Cureus*. 2021;13(11):e19200. doi:10.7759/cureus.19200 | 2, 5 |
|  | Siqueira Santos MM, Sganzerla D, Pereira IJ, et al. Long-Term Mortality and Health-Related Quality of Life After Continuous Versus Intermittent Renal Replacement Therapy in ICU Survivors: A Secondary Analysis of the Quality of Life After ICU Study. *J Intensive Care Med*. 2024:8850666231224392. doi:10.1177/08850666231224392 | 2, 5 |
|  | Smith JM, Lee AC, Zeleznik H, et al. Home and Community-Based Physical Therapist Management of Adults With Post-Intensive Care Syndrome. *Phys Ther*. 2020;100(7):1062-1073. doi:10.1093/ptj/pzaa059 | 0 |
|  | Stavem K, Einvik G, Tholin B, Ghanima W, Hessen E, Lundqvist C. Cognitive function in non-hospitalized patients 8-13 months after acute COVID-19 infection: A cohort study in Norway. *PLoS One*. 2022;17(8):e0273352. doi:10.1371/journal.pone.0273352 | 2 |
|  | Stocking JC, Drake C, Aldrich JM, et al. Outcomes and risk factors for delayed-onset postoperative respiratory failure: a multi-center case-control study by the University of California Critical Care Research Collaborative (UC(3)RC). *BMC Anesthesiol*. 2022;22(1):146. doi:10.1186/s12871-022-01681-x | 2, 4 |
|  | Rousseau A, Colson C, Minguet P, et al. *Characteristics of Mid-Term Post-Intensive Care Syndrome in Patients Attending a Follow-up Clinic: A Prospective Comparison Between COVID-19 and Non-COVID-19 Survivors*. 2023;5. Critical care explorations. | 5 |
|  | Makam A, Burnfield J, Prettyman E, et al. *One-Year Recovery Among Survivors of Prolonged Severe COVID-19: A National Multicenter Cohort*. 2024;52. Critical care medicine. | 0 |
|  | Zangrillo A, Belletti A, Palumbo D, et al. *One-Year Multidisciplinary Follow-up of COVID-19 Patients Requiring Invasive Mechanical Ventilation*. 2021;36. Journal of cardiothoracic and vascular anesthesia. | 5 |
|  | Abbas A, Zayed NE, Lutfy SM. Post ICU syndrome among survivors from respiratory critical illness. A prospective study. *Egypt J Bronchol*. 2019;13(4):505-509. doi:10.4103/ejb.ejb_35_19 | 2 |
|  | Baldwin MR, Pollack LR, Friedman RA, et al. Frailty subtypes and recovery in older survivors of acute respiratory failure: a pilot study. *Thorax*. 2021;76(4):350-359. doi:10.1136/thoraxjnl-2020-214998 | 1, 4 |
|  | Bernard-Valnet R, Favre E, Bernini A, et al. Delirium in Adults With COVID-19-Related Acute Respiratory Distress Syndrome: Comparison With Other Etiologies. *Neurology*. 2022;99(20):e2326-e2335. doi:10.1212/WNL.0000000000201162 | 4 |
|  | Brown SM, Wilson EL, Presson AP, et al. Understanding patient outcomes after acute respiratory distress syndrome: identifying subtypes of physical, cognitive and mental health outcomes. *Thorax*. 2017;72(12):1094-1103. doi:10.1136/thoraxjnl-2017-210337 | 5 |
|  | Carlson CG, Huang DT. The Adult Respiratory Distress Syndrome Cognitive Outcomes Study: long-term neuropsychological function in survivors of acute lung injury. *Crit Care*. 2013;17(3):317. doi:10.1186/cc12709 | 5 |
|  | Denke C, Balzer F, Menk M, et al. Long-term sequelae of acute respiratory distress syndrome caused by severe community-acquired pneumonia: Delirium-associated cognitive impairment and post-traumatic stress disorder. *J Int Med Res*. 2018;46(6):2265-2283. doi:10.1177/0300060518762040 | 5 |
|  | DiSilvio B, Young M, Gordon A, Malik K, Singh A, Cheema T. Complications and Outcomes of Acute Respiratory Distress Syndrome. *Crit Care Nurs Q*. 2019;42(4):349-361. doi:10.1097/CNQ.0000000000000275 | 1 |
|  | Ferrucci R, Dini M, Groppo E, et al. Long-Lasting Cognitive Abnormalities after COVID-19. *Brain Sci*. 2021;11(2). doi:10.3390/brainsci11020235 | 2 |
|  | Ferrucci R, Dini M, Rosci C, et al. One-year cognitive follow-up of COVID-19 hospitalized patients. *Eur J Neurol*. 2022;29(7):2006-2014. doi:10.1111/ene.15324 | 2 |
|  | Gupta E, Jacobs MD, George G, Roman J. Beyond the ICU: Frailty and Post-ICU Disability. Healthcare Use after Acute Respiratory Distress Syndrome and Severe Sepsis. *Am J Respir Crit Care Med*. 2019;199(8):1028-1030. doi:10.1164/rccm.201805-0928RR | 2 |
|  | Hashem MD, Hopkins RO, Colantuoni E, et al. Six-month and 12-month patient outcomes based on inflammatory subphenotypes in sepsis-associated ARDS: secondary analysis of SAILS-ALTOS trial. *Thorax*. 2022;77(1):22-30. doi:10.1136/thoraxjnl-2020-216613 | 2, 5 |
|  | Helms J, Kremer S, Merdji H, et al. Delirium and encephalopathy in severe COVID-19: a cohort analysis of ICU patients. *Crit Care*. 2020;24(1):491. doi:10.1186/s13054-020-03200-1 | 4 |
|  | Holzgraefe B, Andersson C, Kalzén H, et al. Does permissive hypoxaemia during extracorporeal membrane oxygenation cause long-term neurological impairment?: A study in patients with H1N1-induced severe respiratory failure. *Eur J Anaesthesiol*. 2017;34(2):98-103. doi:10.1097/EJA.0000000000000544 | 5 |
|  | Hsieh SJ, Soto GJ, Hope AA, Ponea A, Gong MN. The association between acute respiratory distress syndrome, delirium, and in-hospital mortality in intensive care unit patients. *Am J Respir Crit Care Med*. 2015;191(1):71-78. doi:10.1164/rccm.201409-1690OC | 4 |
|  | Kalra SS, Jaber J, Alzghoul BN, et al. Pre-Existing Psychiatric Illness Is Associated With an Increased Risk of Delirium in Patients With Acute Respiratory Distress Syndrome. *J Intensive Care Med*. 2022;37(5):647-654. doi:10.1177/08850666211019009 | 4 |
|  | Kawakami D, Fujitani S, Morimoto T, et al. Prevalence of post-intensive care syndrome among Japanese intensive care unit patients: a prospective, multicenter, observational J-PICS study. *Crit Care*. 2021;25(1):69. doi:10.1186/s13054-021-03501-z | 5 |
|  | Latronico N, Peli E, Rodella F, Novelli MP, Rasulo FA, Piva S. Three-Month Outcome in Survivors of COVID-19 Associated Acute Respiratory Distress Syndrome. *SSRN Journal*. 2020. doi:10.2139/ssrn.3749226 | 5 |
|  | Latronico N, Peli E, Rodella F, et al. Six-Month Outcome in Survivors of COVID-19 Associated Acute Respiratory Distress Syndrome. *SSRN Journal*. 2020. doi:10.2139/ssrn.3756865 | 5 |
| Note: 0 = Publication form not applicable, 1 = Study design, 2 = Exposure not applicable (or ARDS not clearly described), 3 = Population not applicable, 4 = Outcome not applicable, 5 = Other (e.g. data collection before 2013 or not in English or German) | | |

**SC 5**

**Assessment by Newcastle-Ottawa Scale (NOS)**

| Author (year) | Selection | Comparability | Outcome | Total |
| --- | --- | --- | --- | --- |
| Beaud et al. (2021) | 3 | 2 | 0 | 5 |
| Okazaki et al. (2022) | 2 | 2 | 2 | 6 |
| Pozzi et al. (2023) | 2 | 2 | 2 | 6 |
| Maley et al. (2022) | 2 | 2 | 2 | 6 |
| Martinez et al. (2023) | 2 | 2 | 2 | 6 |
| Monti et al. (2021) | 2 | 2 | 1 | 5 |
| Weidman et al. (2022) | 2 | 2 | 1 | 5 |
| Silvestre et al. (2019) | 2 | 2 | 3 | 7 |
| Rubega et al. (2022) | 2 | 2 | 2 | 6 |
| Sturgill et al. (2023) | 2 | 2 | 1 | 5 |
| Vanginderhuysen et al. (2024) | 2 | 2 | 3 | 7 |
| Duindam et al. (2022) | 2 | 2 | 3 | 7 |
| Jaquet et al. (2022) | 2 | 2 | 2 | 6 |
| Latronico et al. (2022) | 2 | 2 | 2 | 6 |

**SC 6**

**Funnelplots**

- All studies included, p < 0.001 (Egger's test)


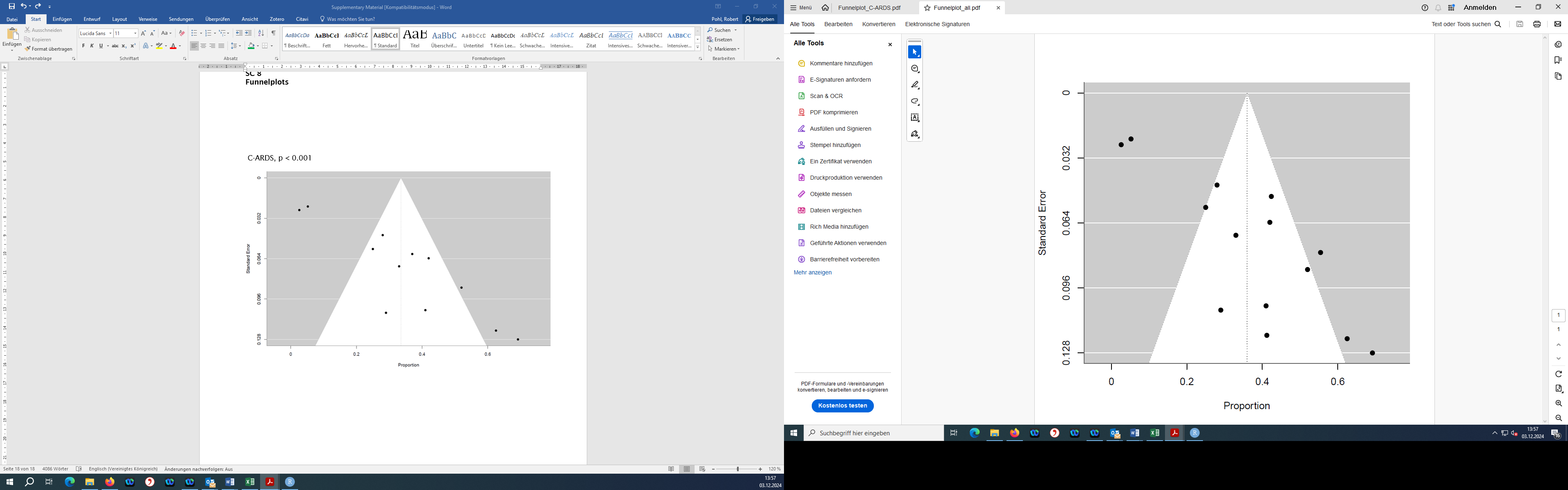


- Studies assessing cognitive impairment in C-ARDS survivors, p < 0.001 (Egger's test)


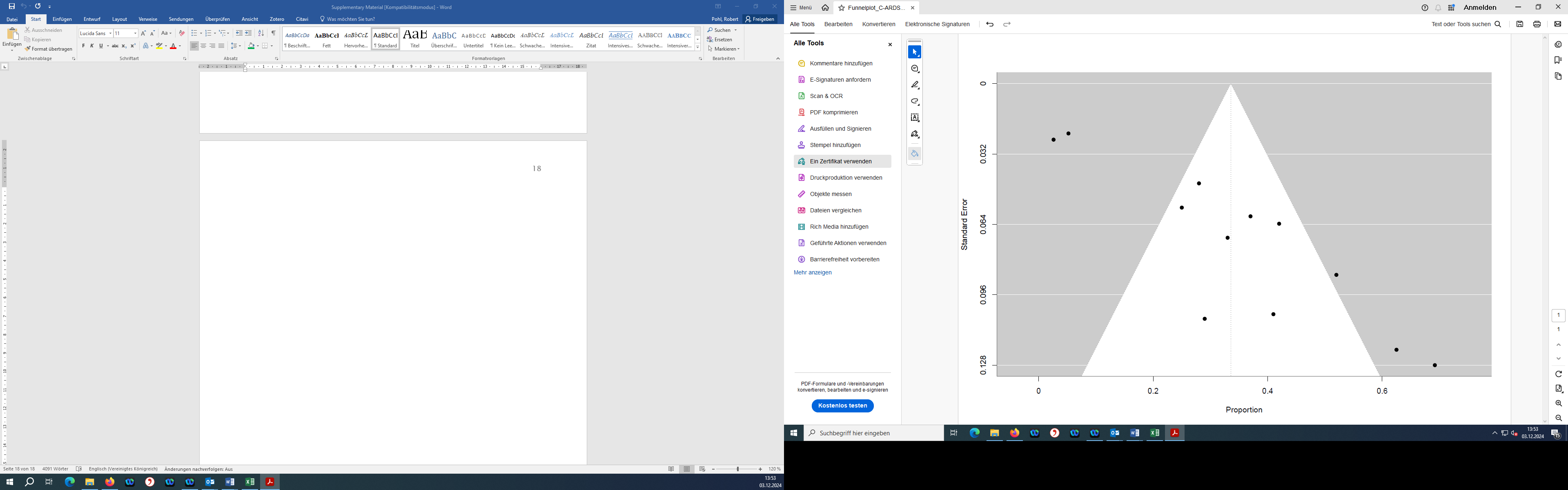


**SC 7**

**Meta-regression scatter plots**


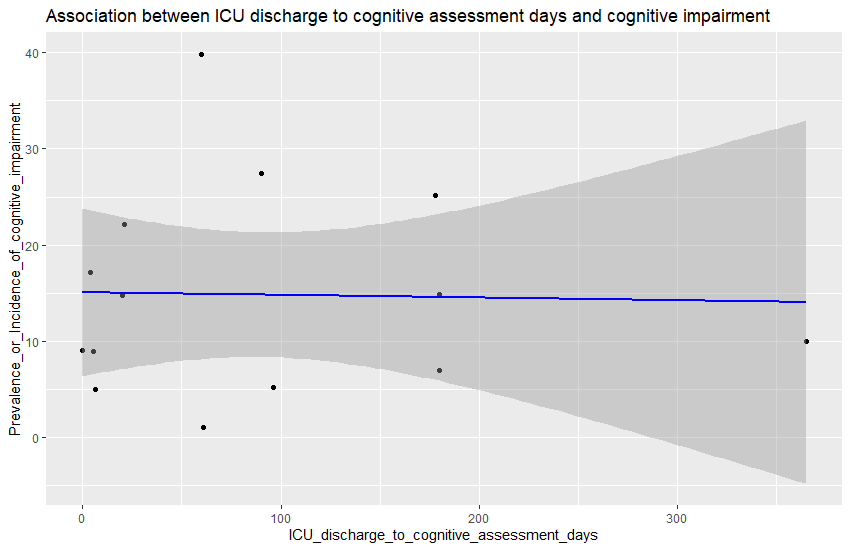

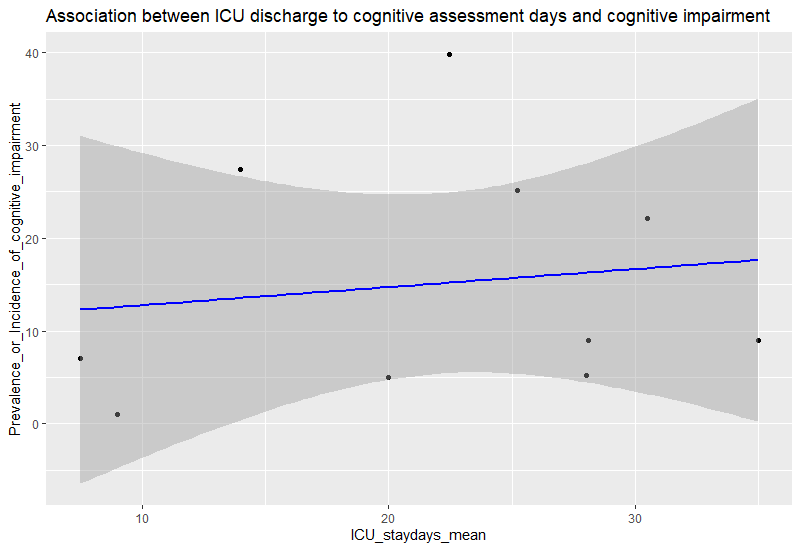


**SC 8**

**Bubble plots**

**
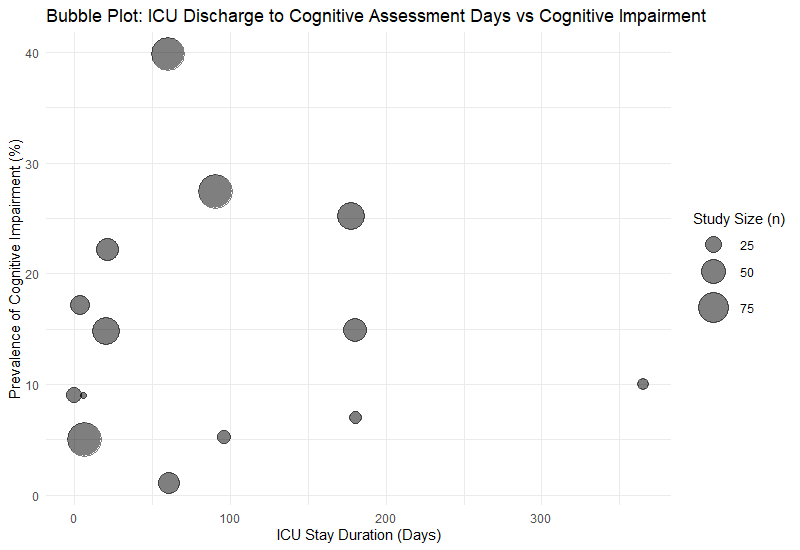
**

**
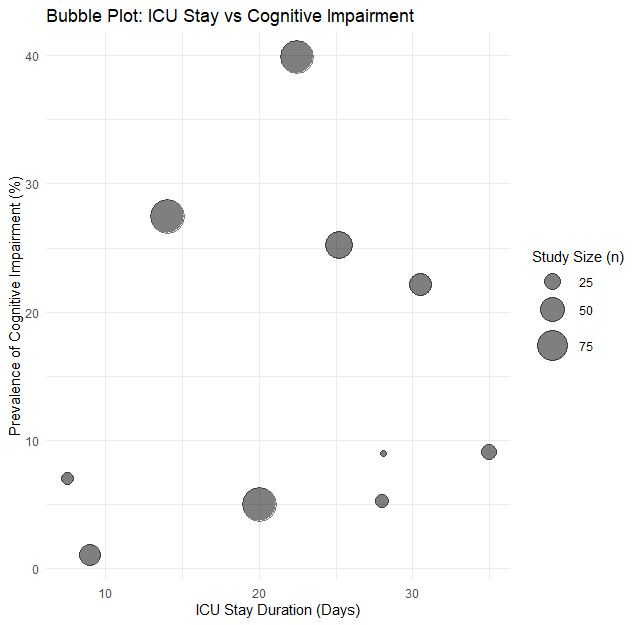
**
